# Sleep Quality and Mental Disorder Symptoms among Canadian Public Safety Personnel

**DOI:** 10.3390/ijerph17082708

**Published:** 2020-04-15

**Authors:** Andréanne Angehrn, Michelle J. N. Teale Sapach, Rosemary Ricciardelli, Renée S. MacPhee, Gregory S. Anderson, R. Nicholas Carleton

**Affiliations:** 1 Anxiety and Illness Behaviours Laboratory, Department of Psychology, University of Regina, Regina, SK S4S 0A2, Canada; 2 Department of Sociology, Memorial University of Newfoundland, Saint John’s, NL A1C 5S7, Canada; 3 Department of Kinesiology & Physical Education, Wilfrid Laurier University, Waterloo, ON N2L 3C5, Canada; 4 Office of Applied Research and Graduate Studies, Justice Institute of British Columbia, New Westminster, BC V3L 5T4, Canada

**Keywords:** public safety personnel, mental disorder symptoms, sleep, mental health, physical health, insomnia

## Abstract

Poor sleep quality is associated with numerous mental health concerns and poorer overall physical health. Sleep disturbances are commonly reported by public safety personnel (PSP) and may contribute to the risk of developing mental disorders or exacerbate mental disorder symptoms. The current investigation was designed to provide estimates of sleep disturbances among PSP and explore the relationship between sleep quality and mental health status. PSP completed screening measures for sleep quality and diverse mental disorders through an online survey. Respondents (5813) were grouped into six categories: communications officials, correctional workers, firefighters, paramedics, police officers, and Royal Canadian Mounted Police (RCMP). Many PSP in each category reported symptoms consistent with clinical insomnia (49–60%). Rates of sleep disturbances differed among PSP categories (*p* < 0.001, ω = 0.08). Sleep quality was correlated with screening measures for post-traumatic stress disorder (PTSD), depression, anxiety, social anxiety disorder, panic disorder, and alcohol use disorder for all PSP categories (*r* = 0.18–0.70, *p* < 0.001). PSP who screened positive for insomnia were 3.43–6.96 times more likely to screen positive for a mental disorder. All PSP reported varying degrees of sleep quality, with the lowest disturbances found among firefighters and municipal/provincial police. Sleep appears to be a potentially important factor for PSP mental health.

## 1. Introduction

Public safety personnel (PSP) face potentially life-threatening risks, long work hours, experience high levels of daily stress, and engage in shift work, which together may make PSP more vulnerable to disordered sleep, mental disorders, and physical disorders relative to the general population [1,2,3,4]. PSP can include boarder services, communications officials (e.g., dispatchers, call center operators), correctional workers, firefighters, paramedics, and police officers [5]. PSP are required to be alert and respond quickly to various demands, yet one-third of police officers in the United States report sleeping less than six hours per night [2]. In a survey of firefighters and paramedics, 93% reported significant sleep disturbances [6]. Among police recruits, sleep duration appears directly associated with psychomotor vigilance [7]. The probability of at least one lapse in performance decreased with every hour spent sleeping; specifically, police recruits had a 56.5% probability of a lapse after sleeping six hours as compared to a 43% probability of a lapse after sleeping for ten hours [8]. PSP appear to have high rates of symptoms consistent with a wide range of mental disorders such as major depressive disorder (MDD), post-traumatic stress disorder (PTSD), general anxiety disorder (GAD), social anxiety disorder, panic disorder, and alcohol use disorder [1]. The increase in compromised mental health among PSP may be associated with exceptionally high rates of exposure to potentially traumatic events [8,9]; however, PSP experiences with sleep may also be a significant contributing factor that remains relatively under researched [10,11].

There is robust evidence of the bidirectional relationship between sleep and mental disorders in the general population [12,13]. Relative to persons without sleep disturbances, those who experience sleep difficulties and insomnia are more likely to report greater physical distress, mental distress, pain, anxiety, and depression, as well as poorer health, social functioning, and quality of life [13,14,15]. Specifically, MDD is associated with disturbances falling and staying asleep, shorter latency in rapid-eye-movement (REM) sleep, decreased delta activity during sleep, and shorter slow-wave sleep [16]; indeed, sleep disturbances (e.g., early awakenings, lower sleep efficiency), both in subjective sleep quality and objective physiological changes, are serious concerns for most people diagnosed with MDD [16]. There is also an established link between PTSD, nightmares, and disturbances in sleep continuity [17]. Abnormalities in sleep patterns, such as decreased delta activity and greater stage-1 sleep, have been identified in persons with PTSD [18]. Individuals with anxiety-related problems also report lower sleep quality, which may impair subsequent daytime functioning and reflexively increase symptoms [19,20]. Nightmares, lower subjective sleep quality, and insomnia are also more commonly reported by persons with suicidal ideation [21], which raises concerns about the compounding effects of symptoms on suicide risk [22].

Insomnia is associated with an increased risk for a wide range of mental disorders, including anxiety and depression, and chronic pain [23]. Researchers reveal that insomnia is an important transdiagnostic factor associated with the development and maintenance of a wide range of mental disorders [12]. There is relatively less research available on how sleep disturbances and insomnia impact PSP mental health, particularly among Canadian PSP, but the available research suggests that there are significant challenges [10,24]. Many police officers report lower sleep quality and getting less sleep than non-officers [10]. Police officers who report greater sleep problems also report greater depressive symptoms [25]. Most paramedics and firefighters report sleep disturbances, which are associated with poorer health, lower job satisfaction [26], increased psychological distress and symptoms of depression [24]. 

There is also little empirical research comparing the diverse effects of PSP occupations on employee sleep and mental health. Shift work, work-related demands, and work dissatisfaction are associated with impairments in functioning, safety, and sleep [26]. Shift work is associated with increased accidents, chronic disease, and lower physical and mental health, regardless of protective factors (e.g., physical activity, only working day shifts) [27]. Among police, hectic schedules (i.e., long work hours and rotating shift work) and routine stressors (e.g., court decisions, discrimination) contribute to fatigue and disturbed sleep [28,29]. Disturbed sleep among police officers can lead to decreased work performance (e.g., administrative errors, falling asleep while driving, safety violations) [30]. Shift work in police officers has been associated with sleep complaints, reduced quality of sleep, and increased stress levels [31]. Long working shifts, rotating shift work, and disturbed sleep has also been associated with mental disorders such as MDD and PTSD among firefighters and paramedics [26,32,33,34]. Additional research on the impact of occupational demands on other types of PSP sleep and mental health could prove useful for preventative initiatives.

The current cross-sectional survey-based investigation was designed to provide the first comparable descriptive estimates of sleep experiences among diverse Canadian PSP and to explore the relationship between sleep quality and mental disorder symptoms. Understanding the current state of Canadian PSP sleep quality across diverse PSP categories, and the possible associated mental health difficulties, can help support and inform tailored mental health initiatives for PSP. Based on previous research: (1) all PSP were expected to report experiencing a number of sleep disturbances [10,26]; and (2) all PSP who screened positive for insomnia were expected to be more likely to screen positive for a mental disorder [34,35].

## 2. Materials and Methods 

### 2.1. Procedure

The current study was approved by the Institutional Research Ethics Board at the University of Regina (IU 2016-107). Canadian PSP were recruited as part of a larger online survey assessing mental health among PSP between September 2016 and January 2017 [1]. Emails were sent to PSP agencies across Canada and the Public Safety Steering Committee (PSSC) of the Canadian Institute for Public Safety Research and Treatment (CIPSRT) to inform individuals of the chance to participate. Links to the survey were also shared across social media websites. Interested participants completed the measures detailed below.

### 2.2. Participants

A total of *n* = 8520 PSP opted to begin answering the survey. A subset of 5813 (32.5% women) respondents answered questions relating to sleep concerns and were included in the current analyses. Respondents were grouped into six public safety categories: communications officials, correctional workers, firefighters (career and volunteer), municipal/provincial police, paramedics, and Royal Canadian Mounted Police (RCMP). The total sample size was sufficient for the planned statistical analyses (see below) based on a power analysis.

### 2.3. Measures

Insomnia Severity Index (ISI) [36]. The ISI is a 7-item self-report measure designed to assess sleep quality (e.g., “How satisfied/dissatisfied are you with your current sleep pattern?”). Each item is rated on a 5-point Likert scale ranging from 0 (very satisfied) to 4 (very dissatisfied). Psychometric analyses of the ISI have revealed good internal consistency (α = 0.91) and convergent validity [34]. Subsequent research with the ISI has replicated the internal consistency, discriminatory capacity, and convergent validity in both community and clinical samples [37]. Use of total score has demonstrated optimal sensitivity and specificity (i.e., a cut-off score of 10 indicating clinically significant distress) [36]. In the current sample, the internal consistency was acceptable for the total score (α = 0.88) and the average inter-item correlation was 0.52.

PTSD Checklist for DSM-5 (PCL-5) [38]. The PCL-5 is a 20-item self-report measure designed to assess symptoms of PTSD (e.g., “Repeated, disturbing dreams of the stressful experience?”). Each item is on a 5-point Likert scale ranging from 0 (not at all) to 4 (extremely). The items assess symptoms over the past month. Psychometric properties for the PCL-5 indicate that it is a reliable and valid measure of PTSD symptoms, with strong internal consistency (α = 0.94) and test–retest reliability (r = 0.82) [39,40,41]. A positive screen for PTSD was determined based on total score (i.e., a cut-off score of 32 used to measure clinically significant symptoms) as well as meeting criteria on each individual symptom cluster [38,42]. In the current sample, the internal consistency was acceptable for the total score (α = 0.96) and the average inter-item correlation was 0.56.

Patient Health Questionnaire (PHQ-9) [43]. The PHQ-9 is a 9-item self-report measured designed to assess symptoms of MDD (e.g., “Little interest or pleasure in doing things.”). Each item is on a 4-point Likert scale ranging from 0 (not at all) to 3 (nearly every day). Items assess the frequency in which participants have been bothered by depressive symptoms in the past two weeks. Psychometric evaluation of the PHQ-9 suggests that it is a valid measure of depression symptoms and severity, with good internal consistency (α = 0.89) and test–retest reliability (r = 0.84) [43,44]. Use of total score provides high specificity and has been recommended for screening purposes (i.e., a total score >9 indicating clinically significant symptoms) [45,46]. In the current sample, the internal consistency was acceptable for the total score (α = 0.90) and the average inter-item correlation was 0.50.

Generalized Anxiety Disorder Scale, 7-Item version (GAD-7) [47]. The GAD-7 is a 7-item self-report measure used to assess symptoms of general anxiety (e.g., “Not being able to stop or control worrying.”). Each item is on a 4-point Likert scale ranging from 0 (not at all) to 3 (nearly every day). Items assess how often participants have been bothered by anxiety symptoms in the past two weeks. Psychometric properties of the GAD-7 suggest that it is a valid measure of anxiety symptoms, with strong internal consistency (α = 0.92) and good test–retest reliability (r = 0.88) [47,48]. Use of the total score provides adequate sensitivity and specificity for discerning the presence of GAD (i.e., a total score >9 can be used to distinguish persons reporting clinically significant anxiety and distress) [49]. In the current sample, the internal consistency was acceptable for the total score (α = 0.92) and the average inter-item correlation was 0.62.

Alcohol Use Disorders Identification Test (AUDIT) [50]. The AUDIT is a 10-item self-report measure designed to assess alcohol consumption, drinking behaviours, and related problems (e.g., “How often do you have six or more drinks on one occasion?”). Each item is measured on a 4-point Likert scale. Items vary in their assessment of frequency (never–daily or almost daily) or intensity (1 or 2–10 or more). Psychometric evaluation of the AUDIT has demonstrated good internal consistency (α = 0.85) and good test–retest reliability (r = 0.83) [51]. Use of total score has demonstrated adequate sensitivity and specificity to be used as a screening tool (i.e., a total score >15 indicating clinically significant hazardous drinking and dependence) [52]. In the current sample, the internal consistency was acceptable for the total score (α = 0.87) and the average inter-item correlation was 0.30.

Social Interaction Phobia Scale (SIPS) [53]. The SIPS is a 14-item self-report measure designed to assess symptoms of social anxiety (e.g., “When mixing socially I am uncomfortable”). Each item is measured on a 5-point Likert scale, ranging from 0 (not at all) to 4 (extremely). The SIPS is comprised of items from the Social Interaction Anxiety and Social Phobia Scales [54]. Psychometric evaluation of the SIPS has demonstrated good internal consistency (α = 0.93), adequate test–retest reliability (r = 0.60), as well as convergent and discriminant validity in large, independent samples [55,56]. Use of the total score has demonstrated adequate sensitivity and specificity in both clinical and community samples (i.e., a total score >20 indicating clinically significant distress) [57,58]. In the current sample, the internal consistency was acceptable for the total score (α = 0.94) and the average inter-item correlation was 0.51.

Panic Disorder Severity Scale (PDSS) [59]. The PDSS is a 7-item self-report measure designed to assess panic disorder symptoms (e.g., “During the past week, how much have you worried or felt anxious about when your next panic attack would occur or about fears related to the attacks?”). Each item is rated on a 5-point Likert scale ranging from 0 (not at all) to 4 (nearly constantly and to a disabling extent). Psychometric evaluations of the measure have demonstrated good reliability (r = 0.71) and internal consistency (α = 0.88) [60]. Use of the total score has demonstrated adequate sensitivity and specificity to be used as a screening tool (i.e., a total score >7 indicating clinically significant symptoms) [56]. In the current sample, the internal consistency was acceptable for the total score (α = 0.93) and the average inter-item correlation was 0.66.

A single item from the Operational Police Stress Questionnaire (PSQ-Op) [61]—“Please indicate how much stress shift work has caused you over the past 6 months”—was used to measure stress related to shift work. The item is rated on a 7-point Likert scale ranging from 0 (no stress) to 6 (a lot of stress).

### 2.4. Analyses

Descriptive statistics were calculated to summarize the amount and quality of PSP sleep. An independent samples t-test was used to determine differences in hours spent sleeping between PSP and the general Canadian population [62]. Analysis of Variance (ANOVA) was used to determine possible differences among PSP categories on insomnia. Post-hoc Tukey’s analyses were used to measure the nature of the differences. Logistic regression analyses were used to determine likelihood of screening positive for a mental disorder if insomnia was present. Pearson correlations were computed to examine the strength of association between mental disorder symptoms and insomnia as well as the association between sleep disturbances and shift work-related stress. 

## 3. Results

### 3.1. Demographic, Sleep, and Mental Disorder Symptom Descriptive Statistics

Demographic information characterizing the sample is presented in Table 1. Most participants were from Western/Central Canada (i.e., British Colombia, Alberta, Saskatchewan, Manitoba), had 10 years or more of experience, and were 39 years of age or older. Most participants were married and had completed some post-secondary education. Information on sleep duration and quality is presented on Table 2. Many PSP in each occupational category reported symptoms consistent with clinical insomnia (see Table 2). PSP reported sleeping on average six hours per night during work nights, and on average seven hours per night during off-shift nights. PSP reported significantly less hours of sleep per work night than the general population t (14,725) = −27.52, *p* < 0.001, Cohen’s d = 0.41. In the most recent Canadian health report on sleep, 25% of Canadian aged 18 to 64 had symptoms consistent with insomnia [63]. In contrast, 55% of PSP in the current sample screened positive for insomnia. Paramedics reported the lowest sleep duration on work nights, while firefighters reported the lowest sleep duration on off-shift nights. Correctional workers reported the highest sleep duration per night on work nights, while communications officials reported the highest sleep duration per night on off-shift nights. PSP reported waking up feeling rested on average two days a week, with the lowest average number of days reported by communications officials, and the highest average number of days reported by firefighters. PSP rated the overall quality of their sleep on average as 2.87 out of 5.00. Almost half of all PSP screened positive for at least one mental disorder based on self-report measure cut-off scores (see Table 2 and Table 3). Communications officials, correctional workers, paramedics, and RCMP screened highest for sleep disturbances, and correctional workers were most likely to be screened positive for mental health problems. Descriptive information on mental disorder self-report measures are presented in Table 3.

### 3.2. Differences in Sleep Quality and Risk for Mental Disorder Symptoms between PSP

Sleep quality differed across PSP categories (Welch’s F (7, 187.77) = 6.37, *p* < 0.01, ω = 0.08) in that RCMP, correctional workers, paramedics and communications officials reported significantly greater sleep disturbances than firefighters and municipal and provincial police. Further, RCMP reported significantly greater sleep disturbances than municipal and provincial police (Post-hoc Tukey’s analyses *p* < 0.05). Logistic regression analyses evidenced that PSP who screened positive for insomnia were 3.43 to 6.95 times more likely to screen positive for another mental disorder (*p* < 0.001; Table 4). Sleep quality was significantly correlated with screening measures for PTSD, depression, anxiety, Social Anxiety Disorder, Panic Disorder, and Alcohol Use Disorder in all PSP categories (r = 0.14–0.71, *p* < 0.01; Table 5). Perceived stress associated with shift work was significantly inversely correlated with duration and quality of sleep (r = −0.06 to −0.22, *p* < 0.01; Table 6).

## 4. Discussion

Sleep difficulties have been commonly documented among certain types of PSP and have been associated with serious work impairments [10,30], poorer physical and mental well-being, as well as psychiatric disorders, e.g., [31]. The current study provides novel estimates of sleep disturbances among different types of PSP and explores the relationship between sleep quality and mental disorder symptoms. The current results can inform opportunities for supporting PSP sleep quality, therein supporting PSP mental health. 

Concerns about sleep disturbances appeared prevalent for all Canadian PSP. Almost half of all participating PSP reported symptoms consistent with clinical insomnia according to self-report measures. The most frequent disturbances as measured by the ISI were found among communications officials, correctional workers, paramedics, and RCMP. Sleep disturbances were significantly associated with numerous mental disorder symptoms, suggesting that sleep quality may be an important factor in PSP mental health. The high prevalence of sleep disturbances reported in the current sample were consistent with previous international research documenting sleep difficulties in firefighters, e.g., [22,31], paramedics, e.g., [26,32,64], and police, e.g., [10,29,31]. Canadian PSP sleep patterns may be impacted by long work hours, varying shift work, high stress, and exposure to potentially psychologically traumatic events [8,9], which may cumulatively increase the risk of mental disorder symptoms [31,65]. Insomnia has been strongly associated with various mental health conditions such as depression, anxiety, and PTSD [23]. In the current study, PSP participants who screened positive for insomnia were approximately 3 to almost 7 times more likely to screen above a clinical threshold for mental disorder symptom measures (i.e., MDD, GAD, PTSD, Social Anxiety Disorder, Panic Disorder, Alcohol Use Disorder). The results are in line with previous police research that demonstrated many officers reported at least one sleep disorder, which was associated with work impairments and safety concerns [31].

Treatment of sleep disturbances, such as insomnia, may have implications for a wide range of mental disorders. Targeting insomnia in treatment may have carry over beneficial outcomes in the improvement of other mental health problems such as depression, PTSD, and anxiety [10]. Sleep quality should be an imperative consideration in treatment settings and workplace policy, particularly regarding the impact of shift work on sleep patterns. Policies restricting the frequency or combination of particularly lengthy shifts (e.g., exceeding 10 h), night shifts, and rotating shift patterns may help mitigate health and performance consequences [24,25,26,66]. A dose–response association between length of shift and sleep disturbances has previously been reported [25]; as such, policies restricting shift duration may increase the safety of PSP and the public. Support from management, organizations, and governments, in consideration of work hours, shift length and shift pattern, sleep timing, and exercise may be pivotal to implement good sleep hygiene behaviours for PSP.

Researchers recently revealed that PSP in general are reluctant to access formal care and that PSP with mental health training report somewhat higher levels of perceived access to care and willingness to access services [67,68]. Therefore, treatment and prevention approaches specifically tailored for PSP may be necessary. A trial program targeting sleep health was well received among firefighters who reported increased awareness and subsequent lifestyle changes [69]. Proper education and the integration of sleep hygiene training (i.e., comprising educational training sessions on healthy sleep and fatigue countermeasures modelled after existing programs [69]) into basic PSP training courses may be essential to maintain PSP health. Individualized support which takes into account PSP occupation, culture, and community may contribute to the greatest concrete change [69]. 

There are several limitations that contextualize the current results and offer direction for future research. First, in the current research, greater stress related to shift work was associated with lower duration and quality of sleep; however, shift work was not objectively assessed. PSP generally engage in shift work [66]; nevertheless, how the patterns of shift work may have influenced our results remains unclear. Future research should empirically assess PSP work schedules to investigate which shift work characteristics are more robustly associated with sleep and mental health disturbances.

Second, the directionality of the relationship between sleep and mental disorders was not assessed in the current research. Insomnia has previously been considered to be a byproduct to mental disorders; however, researchers suggest that the relationship may be better defined as a bidirectional relationship, with sleep disturbances affecting mood, cognition, and quality of life, and acting as a risk or maintenance factor for many mental disorders [12]. Data regarding the onset of sleep problems and mental disorder symptoms was not available in the current sample but should be considered in future research. Longitudinal analysis will provide a better understanding of the directionality of the relationship between sleep and mental disorder symptoms among PSP.

## 5. Conclusions

To our knowledge, the current study is the first to investigate the relationship between PSP occupation, sleep problems, and various mental disorder symptoms. A large number of PSP in each category reported symptoms consistent with clinical insomnia. PSP who had lower sleep quality were approximately 3 to almost 7 times more likely to screen positive for a mental disorder. Rates of sleep disturbances varied among PSP. Firefighters and municipal/provincial police reported the lowest disturbances, while communications officials, correctional workers, paramedics, and RCMP screened highest for sleep disturbances. Further, the results provide an initial comparison between PSP and the general Canadian population. Rates of insomnia endorsed by PSP were more than double those previously reported in the general population. The results highlight the importance of considering sleep management in populations such as PSP who are regularly exposed to both challenging workplace schedules and stressful workplace demands [66]. Workplace policy and initiatives that mandate basic psychoeducation on sleep hygiene while working shift work may prove fruitful in improving sleep quantity and quality for PSP and reducing the risk of PSP mental disorders.

## Figures and Tables

**Table 1:** Demographic Characteristics among Public Safety Personnel in Canada.

Demographic Characteristics	*n* (%)
**Gender**	
Man	3451 (67)
Woman	1695 (33)
**Age**	
18–29	462 (6)
30–39	2122 (27)
39–49	2840 (37)
50–59	1988 (26)
60 and older	314 (4)
**Marital Status**	
Married/common-law	3895 (75)
Single	528 (10)
Separated/divorced/widowed	533 (11)
Remarried	184 (4)
**Ethnicity**	
White	4688 (91)
Other	340 (9)
**Language**	
English	4400 (88)
French	587 (12)
**Education**	
High school or less	458 (9)
Some post-secondary (less than 4 year college/university program)	2721 (54)
University degree/4 year college or higher	1875 (37)
**Province of residence**	
Western/Central Canada (BC, AB, SK, MB)	2700 (53)
Eastern Canada (ON, QC)	1786 (35)
Atlantic Canada (PEI, NS, NB, NFL)	612 (12)
**Public safety personnel category**	
Municipal/provincial police	1279 (26)
RCMP	1283 (26)
Correctional workers	701 (14)
Firefighters	760 (15)
Paramedics	697 (14)
Communications officials	243 (5)

RCMP = Royal Canadian Mounted Police.

**Table 2:** Mean Scores on Sleep Quality Screening Measures and Frequency of Insomnia Positive Screen by Public Safety Personnel Category.

Sleep Quality Screening Measure	Total Sample (5146)	Municipal/Provincial Police (1279)	Royal Canadian Mounted Police (1283)	Correctional Workers (701)	Firefighters (760)	Paramedics (697)	Communications Officials (243)
Positive screen for insomnia	56%	53%	59%	58%	49%	60%	58%
	**M (SD)**						
Insomnia (ISI)	10.78 (6.08)	10.42 (6.20)	11.32 (6.24)	11.20 (6.22)	9.74 (5.72)	11.04 (5.76)	11.27 (5.88)
Hours of sleep work nights	6.22 (1.28)	6.22 (1.24)	6.19 (1.28)	6.32 (1.23)	6.31 (1.30)	6.01 (1.34)	6.19 (1.35)
Hours of sleep off-shift nights	7.24 (1.69)	7.21 (1.56)	7.24 (1.71)	7.42 (1.72)	6.96 (1.53)	7.21 (1.92)	7.57 (1.70)
Days a week feeling rested	2.39 (1.99)	2.51 (2.02)	2.20 (1.97)	2.38 (1.96)	2.67 (2.11)	2.26 (1.91)	2.10 (1.81)
Quality of sleep rating	2.87 (1.03)	2.92 (1.06)	2.77 (1.03)	2.86 (1.02)	2.99 (0.99)	2.84 (1.01)	2.86 (1.04)

ISI = Insomnia Severity Index; quality of sleep measured on a 5-point Likert scale ranging from 1 (lowest) to 5 (highest).

**Table 3:** Mean Scores on Mental Disorder Screening Measures and Frequency of Mental Disorder Positive Screen by Public Safety Personnel Category.

Mental Disorder Screening Measure	Total Sample (5146)	Municipal/Provincial Police (1279)	Royal Canadian Mounted Police (1283)	Correctional Workers (701)	Firefighters (760)	Paramedics (697)	Communications Officials (243)
Positive Screen for Any Mental Disorder	49%	46%	51%	56%	46%	54%	44%
	**M (SD)**						
PTSD (PCL-5)	21.27 (18.80)	18.75 (18.49)	24.94 (20.12)	24.84 (18.86)	16.65 (16.01)	22.35 (18.43)	18.77 (17.70)
Depression (PHQ-9)	6.54 (5.86)	5.61 (5.67)	7.28 (6.06)	7.33 (5.74)	5.34 (5.50)	7.13 (6.05)	7.19 (5.63)
Anxiety (GAD-7)	5.25 (5.01)	4.58 (4.92)	5.95 (5.28)	6.08 (4.97)	4.05 (4.49)	5.71 (4.99)	5.32 (4.81)
Social Anxiety Disorder (SIPS)	10.23 (10.81)	8.31 (9.59)	11.29 (11.58)	12.03 (11.39)	8.03 (9.39)	11.77 (10.90)	11.37 (11.41)
Panic Disorder (PDSS-SR)	2.57 (4.35)	2.02 (3.91)	3.02 (4.72)	3.36 (4.74)	1.66 (3.56)	2.81 (4.51)	2.80 (4.40)
Alcohol Use Disorder (AUDIT)	6.11 (4.96)	6.24 (5.11)	5.53 (4.40)	6.09 (5.20)	7.04 (5.09)	6.15 (5.16)	5.80 (4.88)

PTSD = Post-traumatic Stress Disorder; PCL-5 = Post-traumatic Stress Disorder Checklist for DSM-5; PHQ-9 = Patient Health Questionnaire; GAD-7 = Generalized Anxiety Disorder Scale; SIPS = Social Interaction Phobia Scale; PDSS-SR = Panic Disorder Symptoms Severity Scale, Self-Report; AUDIT = Alcohol Use Disorders Identification Test.

**Table 4:** Logistic Regression for Positive Screens of Any Current Mental Disorder on Insomnia by PSP Group.

PSP Occupation	β	S.E.	Wald	OR	OR 95% CI		*p*
					Lower	Upper	
All PSP	1.55	0.07	523.00	4.71	4.12	5.38	<0.001
Communications Officials	1.67	0.35	23.46	5.31	2.70	10.44	<0.001
Correctional Workers	1.94	0.19	100.45	6.95	4.76	10.16	<0.001
Firefighters	1.41	0.17	69.60	4.11	2.95	5.73	<0.001
Paramedics	1.34	0.18	54.82	3.81	2.68	5.44	<0.001
Municipal/Regional Police	1.23	0.13	87.87	3.43	2.61	4.44	<0.001
RCMP	1.80	0.14	158.69	6.06	4.58	8.02	<0.001

PSP = Public Safety Personnel; RCMP = Royal Canadian Mounted Police.

**Table 5:** Correlations between Insomnia and Mental Disorder Screening Measures Across all PSP.

Mental Disorder Screening Measure	ISI	PCL-5	PHQ-9	GAD-7	SIPS	PDSS
PCL-5	0.58 **	—				
PHQ-9	0.70 **	0.73 **	—			
GAD-7	0.59 **	0.71 **	0.79 **	—		
SIPS	0.42 **	0.51 **	0.56 **	0.56 **	—	
PDSS	0.46 **	0.63 **	0.64 **	0.67 **	0.50 **	—
AUDIT	0.18 **	0.17 **	0.20 **	0.11 **	0.12 **	0.15 **

ISI = Insomnia Severity Index; PCL-5 = Post-traumatic Stress Disorder Checklist-5; PHQ-9 = Patient Health Questionnaire; GAD-7 = Generalized Anxiety Disorder Scale; SIPS = Social Interaction Phobia Scale; PDSS = Panic Disorder Severity Scale; AUDIT = Alcohol Use Disorders Identification Test. ** *p* < 0.001; correlations between total scores on each of the symptom measures.

**Table 6:** Correlations between Sleep Measures and Stress Regarding Shift Work.

Sleep Quality Measure	Shift Work Stress	Hours of Sleep Work Nights	Hours of Sleep Off-Shift Nights	Days a Week Feeling Rested
Hours of sleep work nights	−00.16 **	—		
Hours of sleep off-shift nights	−0.06 **	0.52 **	—	
Days a week feeling rested	−0.20 **	0.39 **	0.20 **	—
Quality of sleep rating	−0.22 **	0.48 **	0.30 **	0.65 **

Quality of sleep measured on a 5-point Likert scale ranging from 1 (lowest) to 5 (highest); shift work stress measured on a 7-point Likert scale ranging from 0 (no stress) to 6 (a lot of stress); ** *p* < 0.001; correlations between single-item questions.
